# Increased Intraocular Pressure and Hyperglycemic Level in Diabetic Patients

**DOI:** 10.1371/journal.pone.0151833

**Published:** 2016-03-22

**Authors:** Maggie B. Hymowitz, Donny Chang, Edward B. Feinberg, Sayon Roy

**Affiliations:** 1 Department of Ophthalmology, Boston University School of Medicine, Boston, Massachusetts, United States of America; 2 Department of Medicine, Boston University School of Medicine, Boston, Massachusetts, United States of America; Cedars-Sinai Medical Center; UCLA School of Medicine, UNITED STATES

## Abstract

**Purpose:**

To determine whether hyperglycemic levels as determined from high hemoglobin A1c (HbA1c) levels influence intraocular pressure (IOP) in patients with non-proliferative diabetic retinopathy (NPDR).

**Methods:**

A retrospective chart review was performed on subjects with a diagnosis of NPDR and a corresponding HbA1c level measured within 90 days before or after an IOP measurement over a two-year period. Exclusion criteria included a diagnosis of glaucoma or treatment with IOP lowering medications or oral or topical steroids.

**Results:**

Using 14.5mmHg as a baseline mean value for IOP, 42 subjects had an IOP < 14.5mmHg and mean HbA1c of 8.1±1.1, while 72 subjects had an IOP ≥ 14.5mmHg and a mean HbA1c of 9.0±2.1. Although there was an overlap in the confidence intervals, a significant difference (P = 0.01) in the mean HbA1c level was observed in regression analysis between the two groups. Importantly, diabetic subjects with elevated HbA1c levels rarely (<1%) exhibited reduced IOP levels.

**Conclusions:**

Diabetic subjects with elevated HbA1c levels exhibited significantly higher IOPs compared to those with lower HbA1c levels. Findings from this study indicate an association between hyperglycemia and elevated IOP and that poor glycemic control may contribute to increased IOP levels in long-term diabetic patients.

## Introduction

Epidemiological data suggests that patients with diabetes are at increased risk of developing primary open angle glaucoma (POAG) [1–6]. Various studies have reported a high prevalence of type 2 diabetes and POAG among different ethnic populations including Australians (6), Japanese [7], and Latino and American women [8, 9]. A recent study also found that the IOP of eyes in patients with uncontrolled diabetes was significantly higher than the IOP of eyes in patients with controlled diabetes [10]. Although the underlying mechanism for this phenomenon remains unclear, in vitro studies conducted in our laboratory suggest that high glucose conditions can induce excess extracellular matrix (ECM) synthesis by trabecular meshwork cells. This may lead to ECM accumulation in the trabecular meshwork, contributing to blockage of aqueous outflow [11, 12]. In the current study, we determined whether an association exists between elevated blood glucose levels and elevated IOP in subjects with NPDR.

## Methods

The Boston University School of Medicine Internal Review Board approved this study. This study was a retrospective chart review. All patient records/infomration was anonymized and de-identified prior to analysis. As such, participants were not required to provide informed consent for their clinical records to be used in this study. The study population consisted of subjects from the Boston Medical Center (BMC) ophthalmology clinic. Age, gender, medical conditions, use of medications, type of diabetes, blood pressure, and time of IOP measurement were documented. Individuals were excluded if they did not have an IOP measured by Goldmann applanation tonometer [13], had a diagnosis of glaucoma, were using or had ever used IOP lowering medications, or were using oral or topical steroids. The cut-off value of 14.5 mmHg for IOP level was chosen based on human and animal studies. A study investigating the efficacy of IOP lowering drugs used IOP level between 14 and 15 mmHg as the mean baseline value [14]. A recent study reported that the mean IOP level measured in 281 healthy adult C57BL/6 mice was 14.5 mmHg [15]. Subjects in this chart analysis were separated into two groups based on IOP level: those ≥14.5mmHg and those <14.5mmHg. The HbA1c values of each group were averaged and expressed as mean ± SD. The results were analyzed by one-way ANOVA, and significance was determined using post hoc comparison test. A value of P<0.05 was considered significant.

## Results

**Fig 1:**
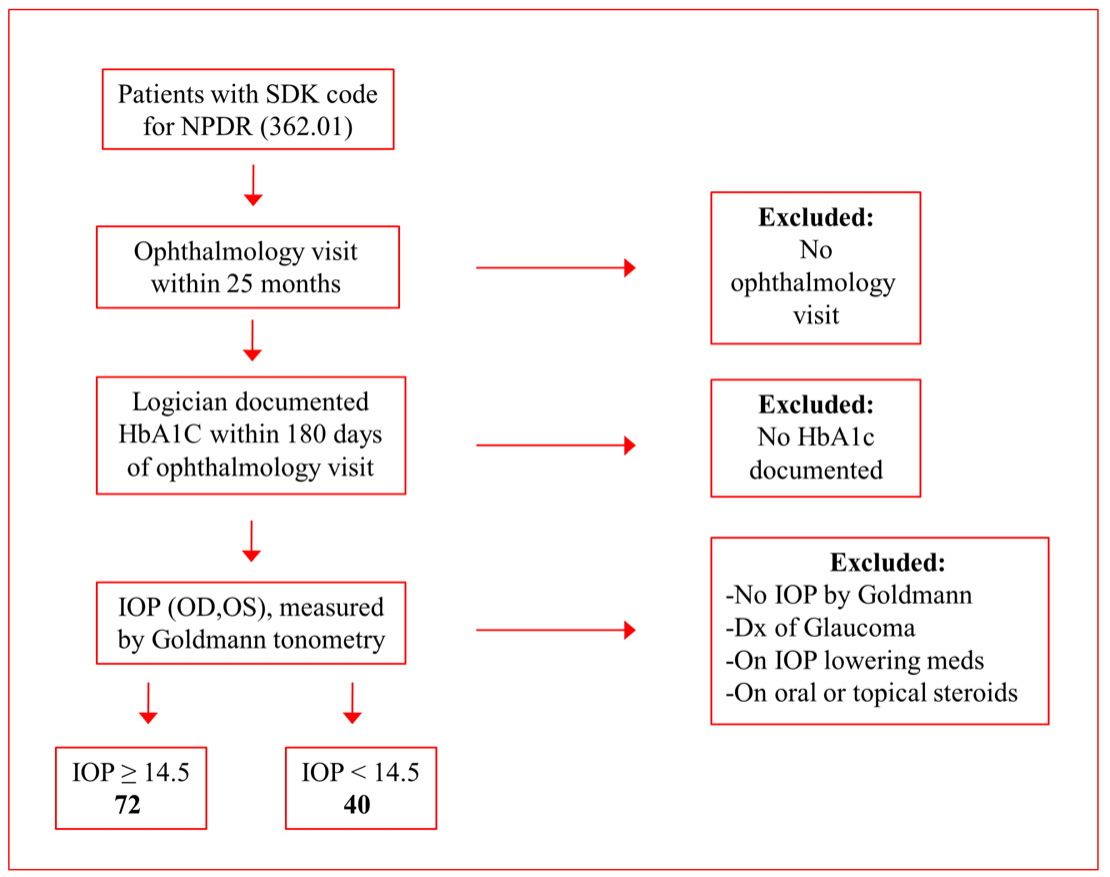
Derivation of study population. The data from diabetic individuals presented in this study were derived based on inclusion or exclusion criteria as shown in the flow chart.

**Fig 2:**
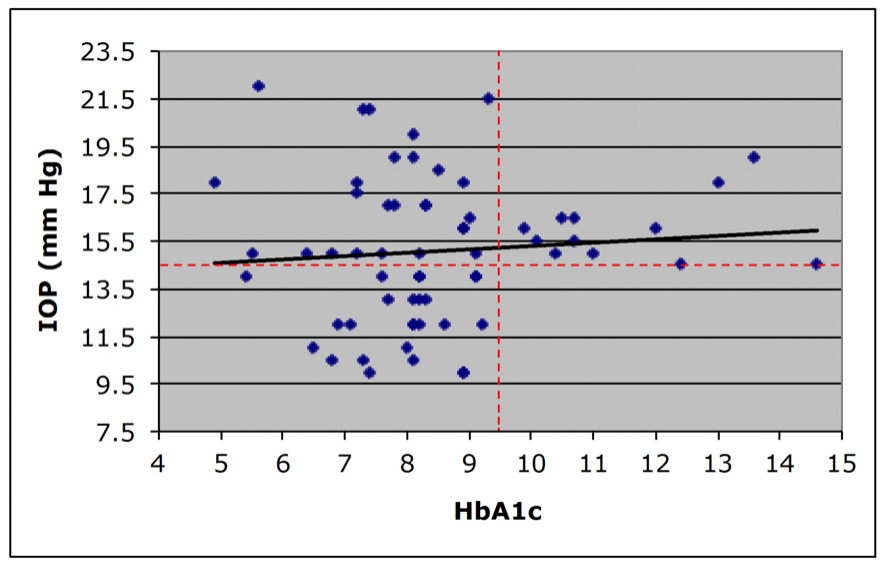
Plot of IOP and HbA1C with regression line representing 114 subjects. The data points representing all subjects are divided into four quadrants (dotted vertical line for HbA1C and dotted horizontal line for IOP). Linear regression plot showing diabetic patients with high HbA1c level rarely exhibit low IOP (lower right quadrant). The solid black line shows linear regression. Note, the horizontal dotted red line represents IOP at 14.5 mm Hg used as proxy population mean value.

A retrospective chart analysis was performed from records of 114 subjects (30% male, 70% female) who met the inclusion criteria for the study. These subjects had a documented diagnosis of Type II diabetes, NPDR noted on dilated fundus exam, and a HbA1c value within the accepted timeframe; the demographic data are presented in Table 1. Fig 1 demonstrates the derivation of the study population. Forty-two subjects had an IOP of <14.5 mmHg, ranging from 10–14 mmHg, with an average HbA1c of 8.1±1.1. Seventy-two subjects had an IOP of ≥ 14.5 mmHg, ranging from 14.5–22 mmHg, with an average HbA1c of 9.0±2.1 (P = 0.01.) Regression analysis (Fig 2) indicated that diabetic subjects with higher HbA1c levels rarely exhibited an IOP less than 14.5 mmHg. An association between IOP and HbA1c levels was noted in patients with HbA1c level at 9.5 and higher but not in patients with HbA1c level below 9.5. This distribution is striking and may relate to how well glycemic level is controlled. Poorly managed glycemic level appears to be associated with elevated IOP. Additionally, this study examining the charts of both male and female diabetic patients suggests no significant difference in the IOP levels between the two genders.

**Table 1:** Distribution of the study population.

Subjects	Low IOP (<14.5)	High IOP (≥14.5)
Gender (F/M)	2.55	2.27
Mean age (yrs)	60.2	58.8
Mean time of IOP measurement	11:26 AM	12:13 PM
Mean HbA1c	8.1±1.1*	9.0±2.1*

Subjects with an IOP of less than 14.5mmHg and those with greater than equal to14.5mmHg have mean HbA1c between the two groups that are significantly different (*p = 0.014).

## Discussion

This is the first report indicating that long-term diabetic patients with elevated HbA1c levels exhibit significantly higher IOPs compared to those with lower HbA1c levels. In particular, findings indicate a statistically significant difference in HbA1c levels between diabetic subjects with low IOP and those with high IOP. In addition to the numerical values from regression analysis, it is particularly striking that it is unlikely for an individual to have a high HbA1c level with a low IOP. Retrospective chart analysis identified only one individual in the study group of 114 subjects who had a high HbA1c and a low IOP (Fig 2; lower right quadrant). It is likely that patients with HbA1c above 9.5 were unable to maintain appropriate clinical/glycemic care. These results suggest an association between elevated glucose levels and increased IOP. In the current study, the exclusion criteria were carefully selected so as not to bias the results. The exclusion of patients with existing glaucoma avoids potential bias toward higher IOPs intrinsic to the diagnosis of glaucoma and not related to HbA1C level. The exclusion of patients using medications that lower IOP or topical or systemic steroids was necessary since these drugs add a potential confounding variable to the relationship between HbA1C and IOP being investigated. Overall, our findings are consistent with other reports in the literature. The Framingham Heart Study reported a positive correlation between blood glucose levels and IOP [1]. The Rotterdam Study [5] and the Wisconsin Study of Diabetic Retinopathy [16] reported higher IOPs in diabetic patients compared to those in the non-diabetic individuals. Additionally, a recent report indicating that poor glucose control in diabetic patients can contribute to increased IOP [10] highlights the possibility of this relationship. While there are reports that suggest outflow facility decreases with age and that aging may increase IOP [17–19], other studies report no age-related IOP increase [20]. In the current study, the mean age of the patients in the two groups are 58.8 years and 60.2 years, as such the issue of IOP dependence on age is not considered a contributory factor.

The underlying mechanism to explain why high blood glucose levels in patients with diabetes may promote increased IOP still remains unclear. The trabecular meshwork through which much of the aqueous drainage occurs represents a specialized tissue composed of various ECM components including fibronectin, laminin, and collagen IV [12, 21, 22]. These ECM components assemble together in a highly organized manner to form a network that is cross-linked, integrated, and scaffold-like. The composition of these ECM components in the trabecular meshwork can influence the meshwork’s ultrastructure and function including maintenance of outflow facility [23]. In a previous study, using an in vitro cell culture model, we have shown that trabecular meshwork cells grown in high-glucose condition upregulates mRNA and protein synthesis of fibronectin, an ECM component [12], and that the excess deposition of ECM components produced by these cells could play a role in the blockage of aqueous outflow through the trabecular meshwork and thereby lead to elevated IOP and the development of POAG [22, 24].

A significant increase in aqueous humor glucose levels of patients with diabetes (3.2 mM vs. 7.8 mM) was reported by Davies et al [25]. Results from a study in our laboratory showed a two- to threefold increase in aqueous humor glucose levels in diabetic rats compared to those of non-diabetic control rats [12]. Since aqueous humor flows out through the trabecular meshwork and is in constant contact with the trabecular meshwork cells, it is likely that the high glucose milieu promotes changes in the trabecular meshwork’s constituents and influences biochemical functions of these cells. The current observation supports the possibility that elevated glucose levels in aqueous humor of diabetic patients may induce increased ECM accumulation in the trabecular meshwork contributing to resistance in aqueous outflow and elevated IOP. This finding suggests an association between high HbA1c and increased ECM synthesis ultimately promoting increased IOP in patients with diabetes. Although the pathogenesis of glaucoma is still not fully understood, the role of high intraocular pressure as a risk factor for glaucoma has been established. Studies indicate that increased IOP is associated with the death of retinal ganglion cells [26, 27], and that optic neuropathy can develop from elevated IOP, which progressively damages optic nerve head due to mechanical compression. Eventually such compression can lead to progressive loss of optic nerve fibers manifested by visual field loss. In the context of the present study, the patients’ IOP levels appear low with tight glycemic control. Sustained hyperglycemia may be a risk factor towards the development of elevated IOP. Although both type I and type II diabetic patients can have sustained hyperglycemic conditions, in this retrospective chart analysis only type II diabetic patients were examined. As such further studies are needed to assess the role of hyperglycemic condition and its effect on IOP in type I diabetic patients. Findings from this study indicate that early HbA1c monitoring may be useful in assessing potential risk for developing increased IOP in patients with diabetes. Further studies are necessary to better understand the association between hyperglycemia and IOP.
